# Chemotherapy with or without estramustine for treatment of castration-resistant prostate cancer: A systematic review and meta-analysis

**DOI:** 10.1097/MD.0000000000004801

**Published:** 2016-09-30

**Authors:** Zhiqiang Qin, Xiao Li, Jianzhong Zhang, Jingyuan Tang, Peng Han, Zhen Xu, Yajie Yu, Chengdi Yang, Chengming Wang, Ting Xu, Zicheng Xu, Qing Zou

**Affiliations:** a Department of Urologic Surgery, The Affiliated Cancer Hospital of Jiangsu Province of Nanjing Medical University; b Department of Urology, The First Affiliated Hospital of Nanjing Medical University, Nanjing, China.

**Keywords:** castration-resistant prostate cancer, chemotherapy, estramustine, meta-analysis

## Abstract

**Background:**

Recently, increasing relevant studies researched the efficacy of castration resistant prostate cancer (CRPC) patients using chemotherapy with or without estramustine, in order to assess the efficacy and toxicity of combining estramustine with chemotherapy for the treatment of CRPC.

**Methods:**

Relevant randomized clinical trials were systematically searched from the databases Pubmed, Embase, and Web of science up to April 1, 2016. Data were centrally extracted and analyzed from the previous studies by 2 independent reviewers. The primary endpoint was overall survival (OS) with pooled hazard ratios. Secondary endpoints were prostate-specific antigen (PSA) response and grade 3 or 4 toxicity using pooled odds ratios. Stata version 12.0 software was used for statistical analysis.

**Results:**

Overall, this meta-analysis identified 9 eligible articles, including a total of 956 patients, who had been accrued between January 1, 1993 and December 1, 2010 and randomly divided into chemotherapy with estramustine and without estramustine. Chemotherapy (with or without estramustine) consisted of docetaxel, paclitaxel, ixabepilone, epirubicin, and vinblastine. Patients who received chemotherapy with estramustine had a better improvement in PSA response rate, comparing those without estramustine (OR = 1.84, 95% CI = 1.20–2.80). However, OS between the 2 groups indicated no significant differences (HR = 0.90, 95% CI = 0.77–1.05). Besides, these results of meta-analysis showed no obvious differences between these 2 groups in grade 3 or 4 adverse effects, including anemia (OR = 0.78, 95% CI = 0.38–1.57), neutropenia (OR = 0.91, 95% CI = 0.59–1.43), thrombocytopenia (OR = 0.68, 95% CI = 0.19–2.42), nausea (OR = 2.34, 95% CI = 0.81–6.72), vomiting (OR = 2.43, 95% CI = 0.69–8.51), diarrhea (OR = 3.45, 95% CI = 0.93–12.76), fatigue (OR = 0.67, 95% CI = 0.32–1.41), neuropathy (OR = 0.54, 95% CI = 0.21–1.44), allergic reaction (OR = 1.60, 95% CI = 0.37–6.84), thromboembolic event (OR = 2.18, 95% CI = 0.86–5.51), and edema (OR = 1.02, 95% CI = 0.18–5.95).

**Conclusions:**

This meta-analysis indicated chemotherapy with additional estramustine increased the PSA response rate. However, OS and grade 3 or 4 toxicity were not improved for these patients with CRPC.

## 1 Introduction

Prostate cancer (PCa) is the most frequently diagnosed malignancy, ranking second as a cause of tumor death among men in the western countries.^[1,2]^ Most patients with advanced PCa are initially sensitive to androgen deprivation. Thus, androgen-deprivation therapy has been the mainstay of first-line treatment for recurrent or metastatic PCa.^[3–5]^ Nevertheless, under prolonged androgen deprivation, patients with PCa invariably become refractory to hormonal manipulation and then gradually have progressed to castration-resistant prostate cancer (CRPC), which metastatic dissemination usually involves the bones.^[6,7]^ Although possible chemotherapeutic strategies for CRPC patients have been constantly increasing, overall survival (OS) benefit cannot worth mentioning, with median survival lasting just 18 months in large phase III randomized trials.^[8,9]^

Estramustine phosphate is a nornitrogen mustard linked to estradiol-1β-phosphate.^[10]^ In addition, estramustine is metabolized into estrone and estradiol after absorption from the gastrointestinal tract, which is able to selectively penetrate into cells of the prostate and prostate tumor metastases. Besides, estramustine not only mainly inhibits microtubule function by binding to both tubulin and microtubule-associated proteins, but also depolymerizes cytoplasmic microtubules, leading to an inhibition of mitosis and induces cell apoptosis by disrupting the nuclear matrix.^[11–14]^ More than anything, single-agent therapy using estramustine in human has little antitumor activity in the treatment of patients with CRPC. However, the microtubule-inhibitory properties of estramustine may lead to the hypothesis that a synergistic antitumor effect can be achieved by combining estramustine with other microtubule inhibitors.

To date, several phases II and III randomized clinical trials (RCTs) have investigated the detailed comparison between chemotherapy with or without estramustine in terms of OS and prostate-specific antigen (PSA) response rate of patients with CRPC. However, these results remained inconsistent or even contradictory. In addition, lack of further research in different level of toxicity systematically illustrated comprehensive understanding of the associations about chemotherapy with or without estramustine in patients with CRPC in some previous meta-analyses.^[15,16]^ Hence, in order to clarify whether estramustine is effective and safe, an updated meta-analysis was conducted by including all individual patient data from eligible studies to identify this statistical evidence.

## 2 Materials and methods

A comprehensive search was conducted among Pubmed, Embase, and Web of Science for relevant articles, covering all the papers published until April 1, 2016, and no language restrictions were applied. The combinations of the following search items were included: “estramustine,” “prostatic neoplasms” or “castration-resistant prostate cancer” and “randomized controlled trial” (“phase III,” “phase II,” and “random”). In addition to electronic search original papers, additional eligible studies were hand-searched from reference lists of original articles or reviews. Besides, we not only contacted the corresponding author to obtain desired information if the research results were unclear or more data were needed, but also asked participating trialists if they were aware of studies not retrieved by the trial search. Furthermore, we also checked abstract booklets and presentations from the annual academic conferences, including American Society of Clinical Oncology (ASCO), European Society for Medical Oncology (ESMO), and European Cancer Organization (ECCO). Meanwhile, the above exposition did not involve an ethical statement.

Studies involved had to meet the inclusion criteria as follows: retrospective phase II and III RCTs were used; the diagnosis of the patients with CRPC was pathologically confirmed; comparison of chemotherapy with or without estramustine for CRPC patients; sufficient data from the included studies could be extracted. The major exclusion criterion was as follows: no available information or complete data; nonoriginal research; duplicates of previous publication.

### 2.1 Data extraction

The identified studies were reviewed carefully by 2 investigators (ZQ Qin and JZ Zhang) independently to determine whether an individual study was eligible for inclusion. The data were centrally extracted from studies involved and the disagreement was solved by a discussion with a third reviewer. All these informations were recorded in a standardized form and the following data were sought from each study: year of publication, first author's name, inclusion period, nationality, ethnicity, study design, number of patients, primary chemotherapy regimen, estramustine dosage, date of birth or age, performance status, serum concentration of PSA, date of randomization, date of last follow-up, survival status, PSA response rate, and any grade 3 or 4 toxicity reaction.

### 2.2 Statistical analysis

All statistical analyses were conducted using Stata software (version 12.0; StataCorp LP, College Station, TX). The analysis was performed on an intention-to-treat basis: the extracted data of each individual were analyzed according to treatment allocated, irrespective of whether they received the treatment of estramustine. The primary endpoint was OS, which calculated from the date of randomization to death for whatever the cause of death, or censored on the date of the last follow-up assessment. The secondary outcomes were PSA response, defined as a decrease in the serum PSA level of ≥50% from baseline and adverse effects of grade 3 or 4 toxicity. Patients involved were censored at their date of death from any cause, or deleted at the date of last follow-up.

For the primary assessment of the efficacy and safety of the addition of estramustine in CRPC patients on OS, we used the overall hazard ratio (HR). Besides, PSA response rate and grade 3 or 4 toxicity were calculated by the pooled odds ratio (OR) with 95% confidence intervals (CIs). When HR of OS could not be extracted from the original articles directly in previous RCTs, we deciphered them from the Kaplan–Meier survival curve as reported by Parmar et al.^[17]^ HR > 1 indicated more deaths or progression in chemotherapy with estramustine group, and OR > 1 reflected more PSA response rate and toxicities in chemotherapy plus estramustine group, and vice versa. The fixed-effects model (a Mantel–Haenszel method) and the random-effects model (a DerSimonian–Laird method) were respectively utilized to pool the data.^[18]^ If existence of heterogeneity was detected, the random-effects model was more appropriate. Heterogeneity assumption was verified by calculating the Chi-square test and *I*^2^ test. After that, subgroup analysis was further carried out by different basic chemotherapy drugs, to appraise sources of heterogeneity. In addition, sensitivity analysis was performed with the method of calculating the results again by omitting 1 single study each time. Besides, Begg funnel plots and Egger linear regression test were taken to examine the publication bias between the studies, and a *P* < 0.05 was considered statistically significant.^[19]^ Two-tailed *P* values were considered statistically significant when less than 0.05.

## 3 Results

### 3.1 Studies characteristics

**Figure 1:**
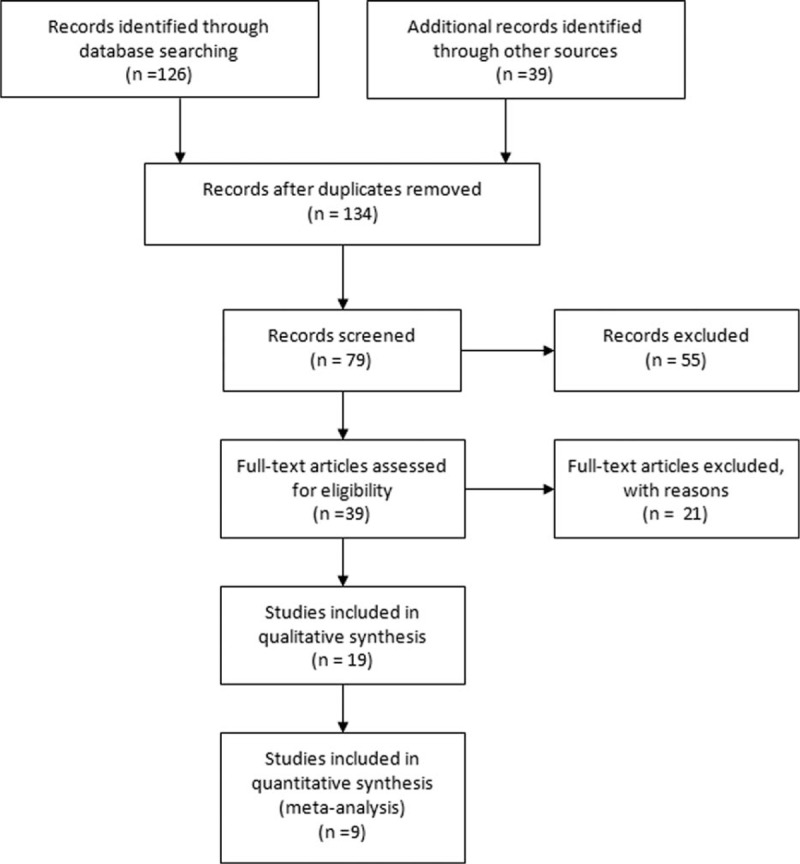
Flow diagram of literature search and selection process.

A total of 9 RCTs including 956 patients met the inclusion criteria and were involved in the current meta-analysis,^[20–28]^ which had been accrued between January 1, 1993 and December 1, 2010. The baseline characteristics of the included studies are listed in Table 1. Chemotherapy (with or without estramustine) consisted of docetaxel (5 trials),^[20–22,24,25]^ epirubicin (1 trial),^[23]^ ixabepilone (1 trial),^[26]^ paclitaxel (1 trial),^[27]^ and vinblastine (1 trial).^[28]^ Moreover, estramustine was given at various doses and schedules in these trials. The flowchart of literature search and screening process is shown in Fig. 1. In addition, due to the limited data provided by the original articles, we respectively removed 2 articles in OS^[22,26]^ and PSA response rate.^[20,25]^

**Table 1:**
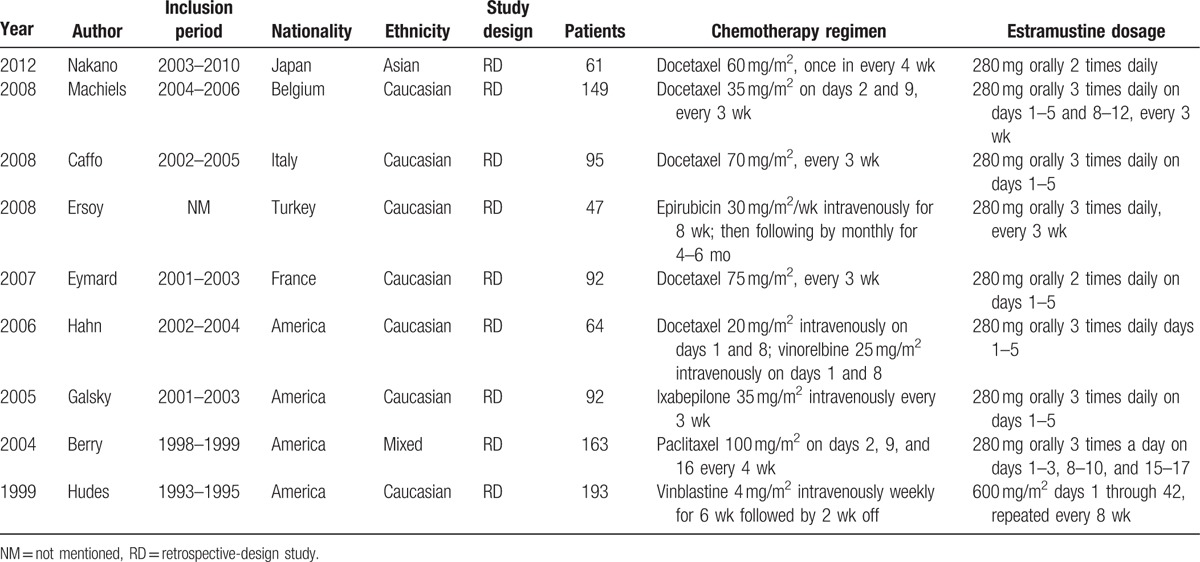
Characteristics of trials assessing chemotherapy with or without estramustine in patients with castration-resistant prostate cancer.

### 3.2 Quantitative synthesis results

The main characteristics of CRPC patients from randomized trials assessing chemotherapy with or without estramustine are collected in Table 2. Overall, median age of patients was 69.35 years old (range: 43–89 years old) in chemotherapy plus estramustine arm, 70.04 years old (range: 41–94 years old) in the chemotherapy without estramustine arm. Besides, median concentration of serum PSA at baseline was 119.6 ng/mL (range: 0.3–8015 ng/mL) in the chemotherapy plus estramustine group, but 106.8 ng/mL (range: 1–5104 ng/mL) in the chemotherapy without estramustine group.

**Table 2:**
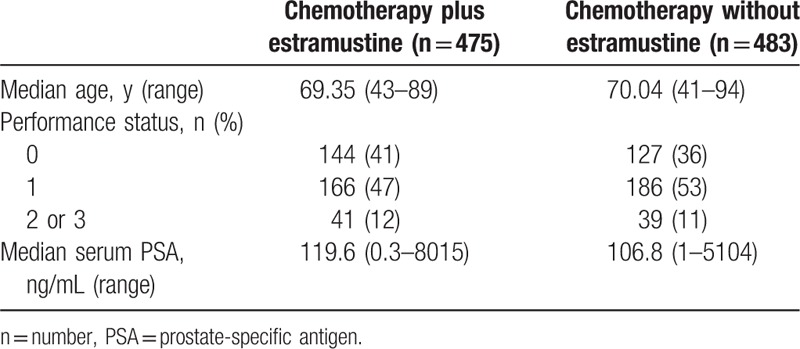
Characteristics of patients with castration-resistant prostate cancer from randomized clinical trials assessing chemotherapy with or without estramustine.

#### 3.2.1 Overall survival

**Figure 2:**
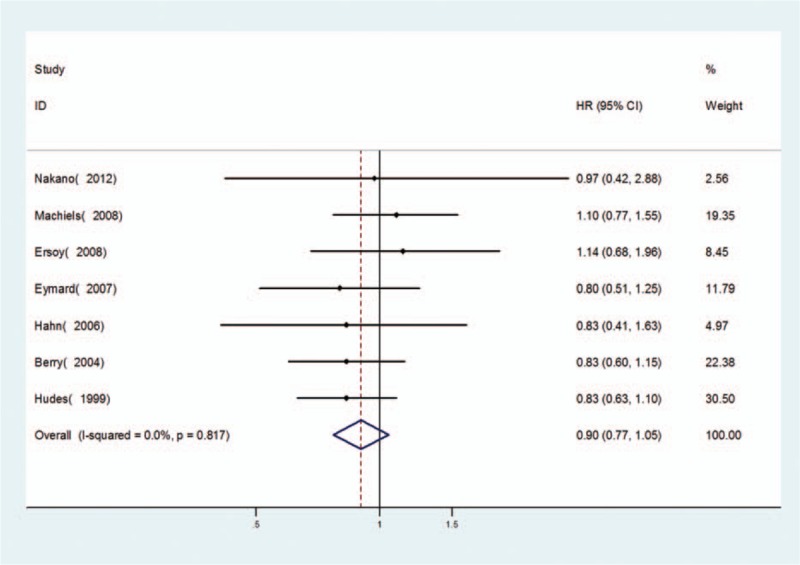
Forest plots of OS associated with chemotherapy with estramustine compared with basic chemotherapy without estramustine in fixed-effects model.

**Figure 3:**
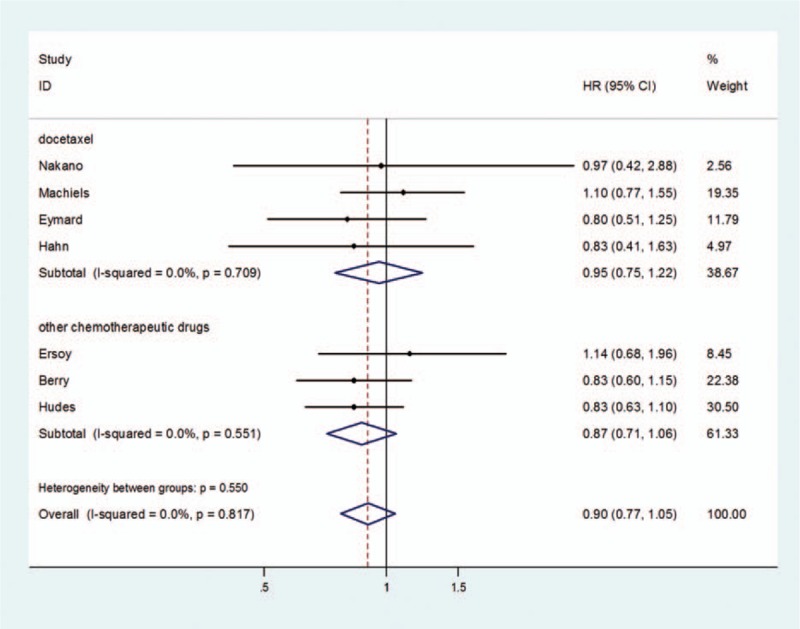
Forest plots of subgroup analysis by different basic chemotherapy drugs of OS associated with chemotherapy with estramustine compared with basic chemotherapy without estramustine.

OS between chemotherapy with and without additional estramustine was no significantly differences in patients (HR = 0.90, 95% CI = 0.77–1.05) (Fig. 2). There was no prominent heterogeneity (*P* = 0.817), and the pooled HR for OS was performed using fixed-effort model. When these studies were stratified by different basic chemotherapy drugs, the results were still no significantly differences whether in docetaxel group (HR = 0.95, 95% CI = 0.75–1.22) or in other chemotherapy regimen group (HR = 0.87, 95% CI = 0.71–1.06) (Fig. 3).

#### 3.2.2 PSA response rate

**Figure 4:**
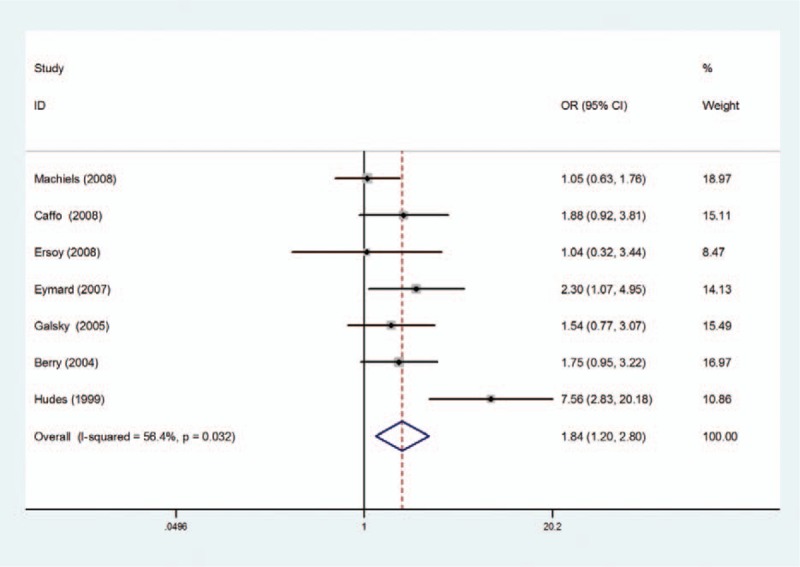
Forest plots of PSA response rate associated with chemotherapy with estramustine compared with basic chemotherapy without estramustine in random-effects model.

However, patients who received chemotherapy plus estramustine had a better improvement in PSA response rate, compared with chemotherapy without estramustine group (OR = 1.84, 95% CI = 1.20–2.80) (Fig. 4). There was obvious significant heterogeneity (*P* = 0.032), which made it necessary to use random-effort model.

#### 3.2.3 Toxicity

In current meta-analysis, the results of grade 3 or 4 toxicity comparing chemotherapy plus estramustine versus chemotherapy without estramustine are shown in Table 3. Outcomes showed that there was no significantly differences in all grade 3 or 4 toxicity, including anemia (4.6% vs 6.0%; OR = 0.78, 95% CI = 0.38–1.57), neutropenia (14.2% vs 15.1%; OR = 0.91, 95% CI = 0.59–1.43), thrombocytopenia (0.8% vs 1.6%; OR = 0.68, 95% CI = 0.19–2.42), nausea (3.6% vs 1.1%; OR = 2.34, 95% CI = 0.81–6.72), vomiting (2.8% vs 0.8%; OR = 2.43, 95% CI = 0.69–8.51), diarrhea (4.5% vs 1.0%; OR = 3.45, 95% CI = 0.93–12.76), fatigue (3.8% vs 6.1%; OR = 0.67, 95% CI = 0.32–1.41), neuropathy (3.0% vs 5.9%; OR = 0.54, 95% CI = 0.21–1.44), allergic reaction (1.5% vs 0.5%; OR = 1.60, 95% CI = 0.37–6.84), thromboembolic event (7.0% vs 2.9%; OR = 2.18, 95% CI = 0.86–5.51), and edema (1.3% vs 1.3%; OR = 1.02, 95% CI = 0.18–5.95).

**Table 3:**
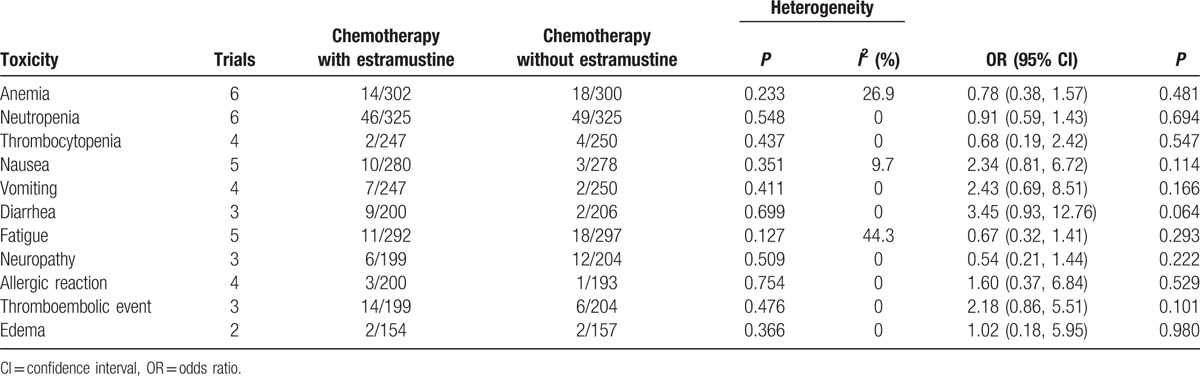
Outcomes of grade 3 or 4 toxicity comparing chemotherapy plus estramustine versus the same chemotherapy without estramustine.

### 3.3 Sensitivity analysis

**Figure 5:**
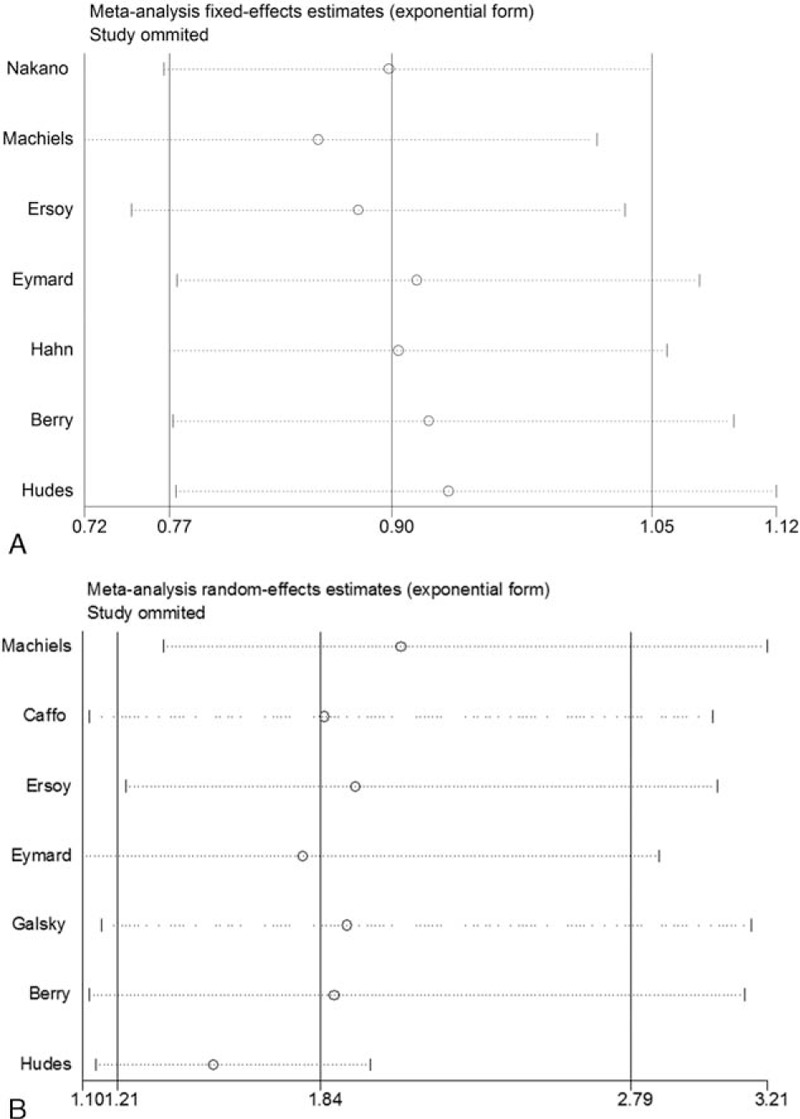
Sensitivity analysis. (A) Pooled HR for OS under fixed-effort model; (B) pooled OR for PSA response rate under random-effort model.

Sensitivity analysis was utilized to detect the influence of each study on the pooled HR or OR by repeating the meta-analysis, while omitting 1 single study each time. The sensitivity analysis for chemotherapy with or without estramustine in the population with CRPC is shown in Fig. 5, demonstrating that no individual study significantly affected the pooled HR or OR. Thus, sensitivity analysis showed that our results were reliable.

### 3.4 Publication bias

The Begg funnel plot was applied to assess the publication bias of the literature, and the shapes of them seemed no evidence of obviously asymmetrical, indicating no significant publication bias, which was also confirmed according to funnel plot (Begg test, *P* = 0.176; Egger test, *P* = 0.321). Therefore, the overall outcomes indicated that our results were statistically robust.

## 4 Discussion

Recently, increasing relevant RCTs researched OS and clinical efficacy of CRPC patients using chemotherapy with or without estramustine, in order to elucidate the significance in chemotherapy with addition estramustine.^[20–28]^ Nevertheless, the outcomes remained inconsistent and controversial. The conflict among them might partially own to the relatively small sample size of individual studies, the different ethnicities and the possible limited effect of individual data in CRPC patients. Moreover, quite a few meta-analyses explored chemotherapy with or without estramustine for CRPC,^[15,16]^ but the results differed a lot. What's more, lack of further research by different stratified analysis in these meta-analyses prevented comprehensive understanding of the disparity and different between 2 groups. Furthermore, additional studies about such distinction have been published since the previous meta-analysis, which might generate great influence on these results. All these factors contribute to the development of the current meta-analysis.

This systematic review and meta-analysis of individual patient data confirmed that, compared with without estramustine, treatment with estramustine markedly increased PSA response rate, but did not improve OS and relieved grade 3 or 4 toxicity reaction in patients with CRPC. As a powerful tool, meta-analysis could provide more reliable results than a single study, especially in explaining controversial conclusions.^[29]^ As a consequence, we took advantage of meta-analysis to clarify the possible benefit when estramustine was added to chemotherapy. To the best of our knowledge, meta-analysis could provide the most comprehensive information by different subgroup analysis. In addition, in the stratified analysis by different basic chemotherapy drugs, the results showed there were no significantly differences in both docetaxel arm and other chemotherapy drugs arm. Thus, the findings of our current meta-analysis suggested that chemotherapy plus estramustine was no superior to chemotherapy without estramustine.

OS is the only validated end point for efficacy in clinical trials investigating patients with CRPC. Seven RCTs reported Kaplan–Meier survival curves in this meta-analysis, and the pooled HR for OS did not show significant differences between 2 chemotherapy with or without estramustine groups. Besides, the limited number of trials, with dissimilar methodologies might affect analysis results. However, chemotherapy of included studies consisted of docetaxel, epirubicin, ixabepilone, paclitaxel, or vinblastine. Moreover, only a limited number of patients treated with the same chemotherapy were available. Therefore, more high-quality RCTs were needed to confirm whether the addition of estramustine to chemotherapy would not improve the OS.

Regarding PSA response rate, as a surrogate for survival after chemotherapy regimen, our analysis suggested that addition estramustine in chemotherapy markedly increased PSA response rate. Whether higher PSA response rate was associated with OS, more RCTs were required to certify such association.

The overall benefit of adding estramustine to chemotherapy should be weighed against the morbidity associated with this drug. However, the optimum estramustine dose and schedule should be used, when combined with chemotherapy drugs was still unclear. In current meta-analysis, the results confirmed that adding estramustine to chemotherapy decreased the risk of chemotherapy-related grade 3 or 4 anemia, neutropenia, thrombocytopenia, fatigue, and neuropathy. Instead, it increased the incidence of chemotherapy-related grade 3 or 4 nausea, vomiting, diarrhea, allergic reaction, thromboembolic event.

Thus, estramustine should be used with caution, since it could increase the incidence of digestive system irritation, thrombosis,^[9]^ and allergic reaction. Among these, thrombosis is one of the most severe complications, difficult to process. In order to prevent estramustine induced thrombotic events, previous researchers had been devoted to find an optimal thromboprophylaxis regimen doses.^[30]^ However, some drugs, including low-dose aspirin, vitamin K, warfarin, and other compounds, were found to be difficult to reduce the incidence of thromboembolic events.^[31]^ Therefore, more efforts should be urgently taken to establish a new standardized anticoagulant therapy of addition of estramustine to maximize the reduction of thrombosis. For another, chemotherapy combined with estramustine showed a significantly low incidence of neutropenia, because treatment with estramustine showed that increasing the leukocyte count led to the myeloprotection in patients with hormone-naive PCa or CRPC.^[32,33]^ However, further studies were needed to clarify this point. Therefore, our meta-analysis indicated that the profile of toxicity associated with both between chemotherapy with estramustine and primary chemotherapy was equivalent, all the toxic side-effects were tolerable and manageable.

Although the overall sufficient and robust statistical conclusions generated from 9 RCTs included in this meta-analysis, some limitations of our study should be taken into consideration when interpreting the data. Firstly, the results were based on unadjusted estimates, with limiting numbers of published studies and insufficient number of patients. As a consequence, inclusion criteria about data of each patient in previous articles were distinction and difference. So as to the relatively high heterogeneity, which could be reduced by subgroup analysis. Secondly, in the stratified analyses, sample size of some subgroups was relatively small, without enough statistical power to explore the efficacy when estramustine was added to chemotherapy compared with the same chemotherapy without estramustine. In addition, as a progressive disease, CRPC results from complex interactions including a variety of genetic and environmental factors, suggesting treatment of CRPC could not be completely influenced by any single drug. Exploring more new and potential therapeutic drugs was required by more researches in the future. What's more, all included trials did not have available data of progression-free survival, and some adverse events were not evaluated in all of the trials. Furthermore, owing to lack of new antiandrogen basic chemotherapy drug, such as enzalutamide and abiraterone in previous studies, further exploration should be done on these aspects in the subsequent researches so that to improve the quality of this study. Last but not least, due to difference of basic chemotherapy regimen, including docetaxel, epirubicin, ixabepilone, paclitaxel, or vinblastine, the efficacy in each individual might be different when estramustine was added to chemotherapy. Accordingly, it was required that further studies could be performed to elucidate the significance of addition of estramustine to chemotherapy if individual data were available.

## 5 Conclusion

The results of the present meta-analysis indicated that chemotherapy with additional estramustine increased the PSA response rate. However, the OS and grade 3 or 4 toxicity were not improved for CRPC patients. As a result, taking into account the current data available in this meta-analysis, patients with CRPC, regardless of the addition of estramustine to chemotherapy and the same chemotherapy without estramustine, could not show any survival benefit in CRPC patients.
